# Fighting from the COVID-19 Frontline: A Junior Doctor’s Perspective on Fear, Duty and Calling

**DOI:** 10.5704/MOJ.2011.001

**Published:** 2020-11

**Authors:** K Rasappan, JYL Oh

**Affiliations:** Department of Orthopaedic Surgery, Tan Tock Seng Hospital, Singapore

**Keywords:** COVID-19 frontline, junior doctor, perspective, burn-out, psychology

## Abstract

As the COVID-19 pandemic ravages the whole world, the frontline clinicians are tirelessly fighting to contain and manage the disastrous effects of the virus from their communities. Stress, despair, fear, physical and psychological burn out, decreased work out put and lowered morale are some side effects this endless battle has had on the frontline healthcare worker. Although there have been many accounts of surgeons working in the frontline, there have only been few reflections on this ongoing battle from the junior clinician’s point of view. In this article, we feature the perspectives of young residents from the orthopaedic unit at the epicenter of the COVID-19 fight in Singapore. We highlight the thoughts, fears, emotions, morale, motivating factors and reflections of junior clinicians while they work at frontlines. Fear in a dangerous new environment and amidst uncertainty is natural. However, a doctor’s call of duty goes far above fear.

## The Apprehension from Sars

Many of the junior doctors from the department, were all in our late teens or just out of high school when SARS hit our nation in 2003^1^. We were ignorant on the impact this deadly virus had on our healthcare system until the first few cases of its much more contagious cousin hit our shores in early 2020. With the first imported COVID-19 case on January 23, 2020^2^, and February 4, 2020 seeing our nation’s first locally transmitted case, hospital management realised that an outbreak was imminent. We saw a staged deterioration in the grim faces of senior doctors who had lived through the trying SARS period. As days progressed, through the cracks that started appearing on their previously composed facades, we increasingly caught glimpses of the mix of panic with streaks of nervousness and fear in the eyes of our seniors. When our stoic head of department (HOD) got choked up when he was sharing about the passing of his close colleague during the SARS fight, there was pin drop silence amongst the juniors. When some of us were scrubbed up for a spine surgery with our senior consultant, who was also the orthopaedic HOD in 2003, with a somber voice, he started sharing some of the stories and the sacrifices of the doctors and nurses then. We could not fathom the devastation SARS had, but knew the impending outbreak was going to be worse.

## Planning the Strike

Hospital management was immediately regrouping to set up coordination and response teams to construct a barricade plan against the impending wave of cases. Hospital policies and the instructions disseminated to the ground kept evolving daily with the only constant being change. With each clinical discipline being assigned a role, juniors in the orthopaedic department were going to be deployed to augment the hospital’s emergency services’ manpower in the screening of high-risk cases^3^. Many of us thought to ourselves, this was what we had signed up for as medical students - to help fellow human beings in need, in a time of crisis!

## The Call for Arms

Along with the excitement of being of service during what we thought was an opportunity of a lifetime, came with it the unfeigned fear. What if we contracted the virus? What if we spread it to our loved ones at home? We soon realised that the fear was universal and palpable. Many of our fellow colleagues had new families with young infants. Some of whom had pregnant wives who were about to deliver or were pregnant themselves. Many were also caring for their aged parents in the same household. Contracting the disease ourselves in the line of work was one thing, but to spread it to family, unthinkable. As we toiled on, doing shift work in the screening center and coming into close contact with suspected and confirmed cases, many of us made arrangements to minimise contact with our loved ones at home by staying instead, at isolated temporary lodging facilities during our high-risk work. Every itchy throat or a heavy cough or sniffle would send alarm bells ringing in our heads. Have we succumbed to the virus? Should we report our symptoms and get ourselves tested? If we get quarantined, our friends and colleagues would have to work harder and our absence would burden them. However, if we didn’t report and were positive, we would do injustice to the hundreds of innocent disease-free patients that we saw. The psychological stress that accompanied each rotation at the screening center and each faint respiratory symptom, bore us down slowly.

There were plans to deploy surgical residents to the intensive care units to augment manpower there. Fear crept in once again due to the unfamiliarity and demands of our potential new job as well as self-doubt in our abilities. However, we knew that we had to stay strong and fight, not only for the cause, but also for the close friend and colleague whom we fought alongside with. It was a privilege to serve the nation during these trying times.

## Sacrifices

All leave except for those on compassionate grounds and maternity leave were frozen indefinitely to conserve manpower for the long fight ahead. All our planned conferences, courses, overseas fellowships and holidays had to be cancelled. The final year residents who were about to sit for their specialist exit exams in April had their exams postponed to later in the year. This meant that our promotion to specialist orthopaedic surgeons also had to be delayed. With the global pandemic not easing, even till today, the senior residents are stuck in limbo as to whether the rescheduled exams are even going to take place. The resultant psychological burn out is rendering them unable to concentrate fully on the current fight, their families or even on their own health.

Training and education for the junior residents has also taken a big blow. Restrictions on physical gatherings, cancellation of traditional teaching/meetings and manpower segregation of various teams had to be implemented to reduce the risk of interhospital virus transmission amongst healthcare staff. Elective surgeries and non-essential clinic visits had to be postponed to conserve hospital resources in an event of a spike in COVID-19 cases as well as to minimise unnecessary contact between patients and providers. Along with the severe drop in surgical workload, came the reduction in cases to learn from. Although the hospital made exceptional arrangements not to delay our residency progression, we started to feel if we would be adequately trained as future surgeons during the prolonged pandemic. Even though innovative methods of E-learning and teleconferencing to facilitate teaching were implemented, we felt that this could not top bedside examination and face to face interaction. Besides, there was nothing like learning surgery other than being scrubbed up for a case.

As morale dwindled through this endless fight, there have been simple yet powerful incidences that have kept us going. Senior surgeons were excused from being deployed at the screening center due to evidence of higher risk of mortality after contracting the disease^4^. Despite being fully aware of the risks, the senior spine consultant some of us was operating with a few weeks ago, volunteered for the deployment. Despite having a multitude of administrative and organisational duties, our current HOD stepped down to volunteer his services on the ground as well. This exceptional leadership by example to ease the stretched manpower situation “flattened the hierarchy”^5^ and instilled confidence amongst the juniors. It built camaraderie and pride. Most importantly, it made us feel that we were all in this together. Effective communication, transparency, welfare and appreciation by our senior management kept us sane, uplifted our morale and strengthened our commitment towards this battle against the invisible enemy.

## Times Ahead

The flipside of the pandemic is that it has taught us so much more having gone through it than without. The use of technology to deliver healthcare to the patients and education to residents without contact; improvisation with limited resources; fortification of systems and changes to workflow to battle the virus; nations working together in solidarity for a common goal etc. The lessons learnt and relationships built will change the practice of medicine and surgery indefinitely. An important lesson from this pandemic thus far that many juniors in our department agree with is that we have to take care of ourselves first before we can help others. Wearing ourselves down will eventually render us giving suboptimal care to our patients. Physical, psychological and emotional well-being of every health care staff needs to be top priority of hospital management. Only if we are well, can we give our one hundred percent to the fight and to our patients.

What motivates us is not the monetary incentives nor the recognition that we are doing something heroic. We draw strength from the whole nation fighting together. Our fellow resident toiling through the graveyard shifts together in the screening center; the taxi driver who risks his life daily fetching strangers around; the hawker who pledges free food and delivers them to the elderly; the cleaners who keep the streets COVID free; the security forces who tirelessly protect the peace of the nation; each citizen staying at home during the semi-lockdown period who is yearning to volunteer in any way to do their part. Our motivation came from the actions of each and every citizen doing their part in this combined battle!

We as healthcare staff around the world continue to toil through this never-ending pandemic, sacrificing much for the greater good. However, we need to draw strength from knowing that the whole world is in this together. We are a resolute bunch who have it within us to never give up. We know that when need arises, when our country beckons our duty, when the common man cries out for help, regardless of discipline or training, all of us are indeed able to step up to duty, far above fear, for the greater calling within us!
